# A Comparison of Clinical, Neonatal, and Maternal Factors Between Early- and Late-Onset Group B Streptococcal Disease

**DOI:** 10.7759/cureus.96857

**Published:** 2025-11-14

**Authors:** Elizabeth A Suschana, Melissa Held, David Banach

**Affiliations:** 1 Infectious Diseases, University of Connecticut, Farmington, USA; 2 Pediatric Infectious Diseases, Connecticut Children's Medical Center, Hartford, USA

**Keywords:** eogbs, gbs, infection prevention, logbs, neonatal sepsis

## Abstract

Background: Universal group B streptococcus (GBS) screening in pregnant people and maternal antibiotic prophylaxis have reduced the incidence of early-onset group B streptococcus (EOGBS) infection. Despite a lower incidence, there is still a high risk of morbidity and mortality without treatment, and there is limited knowledge about late-onset group B streptococcus (LOGBS) infection. Using statewide neonatal sepsis surveillance and hospital admission data, this study describes factors associated with EOGBS and LOGBS.

Methods: This retrospective cohort study combined statewide surveillance data for infants with GBS through Active Bacterial Core surveillance (ABCs) data between 2006 and 2020. Cases were defined as EOGBS (>7 days of age) or LOGBS (7-89 days of age). Chi-square tests and the Mann-Whitney test were used for statistical analysis.

Results: Of the 322 identified cases, 106 (32.9%) were EOGBS, and 216 (67.1%) were LOGBS. LOGBS had a higher rate of meningitis (n = 65, 30.1%) compared to EOGBS (n = 11, 10.4%) (p > 0.001) and had a higher percentage of diagnosis via cerebrospinal fluid (CSF) (n = 51, 23.6%) compared to EOGBS (n = 5, 4.7%; p < 0.001). LOGBS were more likely to receive maternal antibiotics for GBS prophylaxis (n = 129, 61.1%) compared to EOGBS (n = 49, 46.2%; p = 0.012) and more doses of maternal antibiotics (3.39 doses) compared to EOGBS (1.59 doses, p = 0.010).

Conclusions: Differences in epidemiology and clinical manifestations of EOGBS and LOGBS support different approaches to prevent each entity. Strategies for EOGBS prevention include maternal antibiotic prophylaxis and maternal screening. The transmission and pathogenesis of LOGBS are likely different than EOGBS, creating a need for different preventative strategies. Potential mechanisms to prevent LOGBS include maternal vaccination.

## Introduction

In the United States, neonatal bacterial sepsis is a leading cause of infant mortality [1]. Group B streptococcus (GBS) is a leading cause of early-onset group B streptococcus (EOGBS) and late-onset group B streptococcus (LOGBS) sepsis, occurring in 0.2-0.38 cases per 1000 live births and 0.18-0.4 cases per 1000 live births, respectively [1]. Invasive GBS disease, including sepsis and meningitis, has a high disease burden and remains an important cause of illness and death among infants [2-4]. Universal GBS screening during pregnancy and maternal antibiotic prophylaxis have reduced the incidence of EOGBS [4]. Despite a lower incidence, there is still a high risk of morbidity and mortality without treatment [5]. Compared to EOGBS, the incidence of LOGBS has remained relatively stable, suggesting that the transmission of LOGBS may not be affected by maternal intrapartum antibiotic administration [4,6].

Risk factors for EOGBS, including intrauterine distress, maternal GBS colonization, chorioamnionitis, the premature rupture of membranes (ROM), prematurity, very low birth weight, and maternal infection, are well documented in the literature [5]. LOGBS risk factors are less defined but include young maternal age, HIV infection, Black race, prematurity, low birth weight, and maternal colonization [2,7].

This study aims to identify differences in clinical features and maternal/neonatal factors between EOGBS and LOGBS to inform prevention strategies using data from neonatal sepsis surveillance and hospital admissions. Understanding factors associated with LOGBS compared to EOGBS may aid in better classifying risk for LOGBS and the implementation of preventative strategies. Considering the different transmission mechanisms of EOGBS compared to LOGBS, we anticipate that there will be differences in clinical characteristics of the disease, including disease severity, syndrome associated with GBS such as meningitis or bacteremia, and maternal treatment. This article was previously presented as a meeting poster at the 2025 Connecticut Infectious Disease Society meeting on May 15, 2025.

## Materials and methods

The Connecticut Department of Public Health (CT DPH) conducts statewide surveillance for infants with GBS as part of the Centers for Disease Control and Prevention’s Active Bacterial Core surveillance (ABCs) and Emerging Infections Program. Neonatal cases of GBS (<90 days of age) in 2006-2020 were identified through statewide active laboratory surveillance at all acute care hospitals in Connecticut. Standardized case report forms were completed for all incident cases identified. Exclusion criteria from risk factor analysis included the outcome of neonatal death and neonatal age greater than 90 days of life. Cases with missing variable data were excluded from the analysis of that variable but not excluded from the entire study. Cases that did not have criteria for cross-matching, including first name, last name, date of birth (DOB), and/or medical record number, were excluded.

Cases were categorized as EOGBS (<7 days of age) or LOGBS (7-89 days of age) based on the date of positive culture for GBS. Variables include maternal, neonatal, and clinical characteristics. Preterm delivery was defined as <37 weeks’ gestation. Maternal characteristics include demographics, advanced maternal age, and maternal intrapartum antibiotic administration. Neonatal characteristics include demographics, clinical syndrome related to GBS, gestational age, preterm versus term delivery, and birth weight. Clinical characteristics included the rupture of membranes, the rupture of membranes before cesarean delivery, and delivery type. Variables including advanced maternal age, preterm versus term delivery, birth weight, and low birth weight versus normal birth weight were derived from existing variables on the case forms. All other variables were prespecified on the case report form.

Chi-square tests were used to analyze maternal, neonatal, and clinical variables. A Mann-Whitney test was used to analyze the length of stay, doses of maternal intrapartum antibiotics, and maternal age. P-values of <0.05 were considered statistically significant. Variables with a cell count of <10 were consolidated or not included within the analysis. Unknown values were excluded from analysis. Analyses were performed using SPSS v.28 software (IBM Corp., Armonk, NY). The study was approved by the CT DPH Human Investigation Committee and qualified as exempt by the University of Connecticut Institutional Review Board (IRB).

## Results

Three hundred twenty-two cases of GBS were identified. Of these cases, 106 (32.9%) were EOGBS, and 216 (67.1%) were LOGBS. Rates of preterm delivery were similar among EOGBS and LOGBS, 42.5% (n = 45) and 43.5% (n = 94), respectively (Table 1). Among cases of GBS, 26 (8.8%) died during the index admission. EOGBS mortality rate was 13% (n = 14), with a rate of 24% in preterm infants (n = 11) and 5% in term infants (n = 3). Mortality rate in LOGBS was 5.8% (n = 12), with a rate of 6% (n = 6) in preterm infants and 5% (n = 6) in term infants.

**Table 1:** Comparison of infant demographics and neonatal clinical characteristics between EOGBS and LOGBS *A significant p-value. P-value calculated using chi-square tests ^A^Values inside the parentheses are percentages ^B^P-value for univariate comparison of White to other races ^C^Bacteremia without meningitis included bacteremia alone and bacteremia with other syndromes, including cellulitis, pneumonia, arthritis, osteoarthritis, and pericarditis ^D^Meningitis with and without bacteremia also included combinations of meningitis with other clinical syndromes, including pneumonia ^E^Cases with a positive CSF culture and a positive blood culture or other positive culture sites were classified as a positive CSF culture. Cases with a positive blood culture and other positive culture sites were classified as positive blood culture. Other culture sites include pericardial, perineal, and joint. If no culture data was available for a case, the culture was determined based on the clinical diagnosis of bacteremia or meningitis LOS, length of stay; GBS, group B streptococcus; CSF, cerebrospinal fluid; EOGBS, early-onset group B streptococcus; LOGBS, late-onset group B streptococcus

Variable	Total Population (n = 322)	Early Onset (n = 106)	Late Onset (n = 216)	P-value*
Sex				
Female	169	55 (51.9)^A^	114 (52.8)	0.880
Male	153	51 (48.1)	102 (47.2)	
Ethnicity				
Hispanic	104	31 (44.9)	73 (48.3)	0.638
Not Hispanic	116	38 (55.1)	78 (51.7)	
Race				
White	133	47 (58.8)	86 (58.5)	0.971^B^
Black	76	28 (35.0)	48 (32.7)	
Asian/Pacific Islander	16	4 (5.0)	12 (8.2)	
Multiple	2	1 (1.25)	1 (0.6)	
Insurance				
Public	155	44 (47.3)	111 (56.6)	0.138
Private	134	49 (52.7)	85 (43.4)	
Average LOS from infection to discharge (days)				
	28.98	26.55	30.18	0.0459*
Clinical syndrome related to GBS				
Bacteremia without meningitis^C^	230	93 (89.4)	137 (67.8)	<0.001*
Meningitis with and without bacteremia^D^	76	11 (10.6)	65 (32.2)	
Culture type^D^				
Blood^E^	255	98 (95.1)	157 (75.5)	<0.001*
CSF^E^	56	5 (4.9)	51 (23.6)	
Gestational age				
<27 weeks	50	22 (20.8)	28 (13.0)	
28-31 weeks	31	6 (5.7)	25 (11.6)	
32-33 weeks	17	4 (3.8)	13 (6.0)	
34-36 weeks	41	13 (12.3)	28 (13.0)	
37-38 weeks	70	17 (16.0)	53 (24.5)	
39-40 weeks	92	34 (32.1)	58 (26.8)	
41+ weeks	21	10 (9.4)	11 (5.1)	
Preterm versus term delivery				
Preterm (<37 weeks)	139	45 (42.5)	94 (43.5)	0.238
Term (>37 weeks)	183	61 (57.5)	122 (56.5)	
Birth weight				
Extremely low	49	20 (18.9)	29 (13.5)	
Very low	27	6 (5.7)	21 (9.8)	
Low	51	14 (13.2)	37 (17.2)	
Normal	182	63 (59.4)	119 (55.3)	
High	12	3 (2.8)	9 (4.2)	
Low birth weight compared to normal birth weight				
<2500 g	127	40 (37.7)	87 (40.5)	0.638
>2500 g	194	66 (62.3)	128 (59.5)	

Bacteremia was the most common presentation (89.4%, EOGBS; 67.8%, LOGBS). LOGBS had a higher rate of meningitis (n = 65, 30.1%) compared to EOGBS (n = 11, 10.4%) (p < 0.001) (Table 1). LOGBS had a higher percentage of diagnosis via cerebrospinal fluid (CSF) (n = 51, 23.6%) compared to EOGBS (n = 5, 4.7%; p < 0.001) (Table 1). Six (7.9%) infants with GBS meningitis died, with all deaths occurring among those with LOGBS. Among those with bacteremia, 20 (26.3%) died.

GBS bacteriuria during pregnancy was infrequent in EOGBS (n = 14, 14.4%) and LOGBS (n = 29, 14.5%). Rates of maternal GBS colonization in a prior pregnancy were low in EOGBS (n = 2, 1.9%) and LOGBS (n = 3, 1.4%). GBS maternal colonization screening rates were similar in EOGBS (70.8%) and LOGBS (68.8%). GBS colonization screening prior to maternal admission for preterm deliveries was 37.1% (n = 58) with similar rates among EOGBS and LOGBS, 38.6% (n = 17) and 42.7% (n = 41) (p = 0.650), respectively. Rates of GBS colonization screening after maternal admission were higher in LOGBS (n = 56, 25.9%) compared to EOGBS (n = 19, 18.3%). EOGBS had a higher rate of positive screening results after maternal admission (n = 17, 89.5%) compared to LOGBS (n = 21, 38.2%) (p < 0.001). EOGBS was more likely to have a spontaneous rupture of membranes (ROM) (n = 75, 71.4%) compared to LOGBS (n = 111, 52.1%; p = 0.001) and a duration of ROM of >18 hours (n = 26, 25.2%) compared to LOGBS (n =26, 12.4%; p = 0.004) (Table 2).

**Table 2:** Comparison of maternal clinical characteristics between EOGBS and LOGBS *A significant p-value. P-value calculated using chi-square tests ^A^Values inside the parentheses are percentages ^B^Vaginal deliveries included vaginal delivery, vaginal delivery after previous C-section, forceps-assisted delivery, and vacuum-assisted delivery ^C^Cesarean section deliveries included primary cesarean section and repeat cesarean section EOGBS, early-onset group B streptococcus; LOGBS, late-onset group B streptococcus

Variable	Total Population (n = 322)	Early Onset (n = 106)	Late Onset (n = 216)	P-value*
Type of rupture of membranes (ROM)				
Spontaneous	186	75 (71.4)^A^	111 (52.1)	0.001*
Artificial	132	30 (28.6)	102 (47.9)	
Duration of ROM of >18 hours?				
Yes	52	26 (25.2)	26 (12.4)	0.004*
No	260	77 (74.8)	183 (87.6)	
If <37 weeks, ROM before the onset of labor?				
Yes	101	29 (52.7)	37 (33.0)	0.014*
No	66	26 (47.3)	75 (67.0)	
Delivery type				
Vaginal^B^	151	51 (60.0)	100 (55.2)	0.466
Cesarean^C^	115	34 (40.0)	81 (44.8)	
If delivery by cesarean, did ROM happen before cesarean?				
Yes	72	38 (80.9)	34 (38.2)	<0.001*
No	64	9 (19.1)	55 (25.5)	
Average maternal age (years)				
	27.90	27.75	27.97	0.658
Advanced maternal age (>35 years)				
Yes	49	19 (17.9)	30 (14.0)	0.507
No	272	87 (82.1)	185 (86.0)	

Mothers of infants with LOGBS were more likely to have received intrapartum antibiotics (n = 129, 61.1%) compared to EOGBS (n = 49, 46.2%; p = 0.012) (Table 3). The primary intrapartum antibiotic administered was penicillin (EOGBS, n = 23, 46.9%; LOGBS, n = 88, 43.1%) (Table 3). The average number of antibiotic doses in LOGBS was 3.39 compared to 1.59 in EOGBS (p = 0.010) (Table 3). Mothers of infants with LOGBS were more likely to receive antibiotics for GBS prophylaxis (n = 70, 66.0%) compared to EOGBS (n = 17, 16.0%; p = 0.014) (Table 3). The most common indication noted for maternal intrapartum antibiotic administration in EOGBS was “other reasons” (n = 25, 84.0%), including cesarean prophylaxis, mitral valve prolapse prophylaxis, and suspected amnionitis (Table 3).

**Table 3:** Comparison of maternal antibiotic administration characteristics between EOGBS and LOGBS *A significant p-value (<0.05) that was calculated using chi-square tests **A significant p-value (<0.05) that was calculated using Mann-Whitney tests ^A^Values inside the parentheses are percentages ^B^Other reasons for intrapartum antibiotics include cesarean section prophylaxis, mitral valve prophylaxis, and suspected amnionitis ^C^Cephalosporins included cefazolin, cefotaxime, ceftriaxone, and cefoxitin ^D^Penicillins included penicillin, ampicillin, and ampicillin/sulbactam ^E^Other antibiotics administered included vancomycin and gentamicin ^F^Other antibiotics included cephalosporins, vancomycin, and gentamicin GBS, group B streptococcus; EOGBS, early-onset group B streptococcus; LOGBS, late-onset group B streptococcus

Variable	Total Population (n = 322)	Early Onset (n = 106)	Late Onset (n = 216)	P-value
Were maternal antibiotics given?				
Yes	178	49 (46.2)	129 (61.1)	0.012*
No	139	57 (53.8)	82 (38.0)	
Reason for maternal antibiotics^B^				
GBS prophylaxis	87	17 (16.0)	70 (66.0)	0.014*
Others	61	25 (84.0)	36 (24.0)	
Primary maternal antibiotic administered^D^				
Penicillins^D^	113	23 (46.9)	90 (69.8)	
Cephalosporins^C^	42	14 (28.6)	28 (21.7)	
Others^E^	23	12 (24.5)	11 (8.5)	
Penicillin versus other intrapartum antibiotics				
Penicillins^D^	113	23 (46.9)	90 (69.8)	0.005*
Other antibiotics^F^	58	26 (53.1)	39 (30.2)	
Average number of doses of intrapartum antibiotics				
	2.89	1.59	3.39	0.010**

## Discussion

In our study, the total mortality rate was consistent with previously reported mortality ranges of 4%-10% [8,9]. The mortality rate for infants with EOGBS was higher in term infants (5%) compared to a prior reported rate of 2.1% and in preterm infants (24%) compared to a prior reported rate of 19.2% [10]. The mortality rate for LOGBS was lower for preterm infants (6%) compared to a prior reported rate of 7.8% but higher in term infants (5%) compared to a prior reported rate of 3.4% [10].

Our data showed similar factors associated with EOGBS and LOGBS, including maternal colonization, low birth weight, and preterm delivery [2,7]. Rates of GBS colonization screening were similar between EOGBS and LOGBS prior to admission. We identified several differences in maternal and intrapartum characteristics between EOGBS and LOGBS. During admission for labor, mothers of patients with EOGBS were more likely to screen positive, supporting that the transmission and pathogenesis of EOGBS is different than LOGBS [3]. Risk factors for EOGBS include maternal infection and the premature rupture of membranes [5]. In our study, the spontaneous rupture of membranes was more highly associated with EOGBS. Previous studies report that factors predictive of EOGBS, such as the duration of membrane rupture, are not predictive for LOGBS, which is consistent with our study where EOGBS had a significantly higher rate of prolonged rupture of membranes [10].

The recommended antibiotic for prophylaxis for GBS in pregnancy is penicillin [11]. In our study, mothers of infants with EOGBS were less likely to receive intrapartum antibiotics compared to mothers of infants with LOGBS and were more likely to receive other types of antibiotics, such as cephalosporins. These findings are consistent with the current thought that EOGBS rates have decreased due to prophylactic antibiotics [10]. The pathogenesis of LOGBS in neonates is not well defined. Possible mechanisms of transmission include mucosal colonization acquired in the perinatal and postpartum period after prophylactic antibiotics are discontinued, vertical transmission through breast milk, and horizontal transmission from nosocomial and other community sources [12-14]. LOGBS were more likely to receive maternal antibiotics for GBS prophylaxis, more doses of antibiotics, and penicillin compared to EOGBS, suggesting that maternal antibiotic prophylaxis does not impact LOGBS disease. Other strategies to prevent LOGBS warrant exploration, including maternal vaccination, which may result in antibody production, the transmission of antibodies through breast milk, and increased infant immunity to LOGBS. The development of GBS vaccines across decades of research has demonstrated evidence of immunogenicity and safety but never resulted in a licensed product. Currently, two vaccine candidates are in the advanced stages of clinical development [13,14].

Infants with EOGBS were more likely to have a positive blood culture. Infants with LOGBS had higher rates of CSF-positive cultures and clinical findings of meningitis, consistent with current findings of higher rates of meningitis in LOGBS than EOGBS [10]. In our study, LOGBS complicated by meningitis had a higher mortality rate compared to other clinical syndromes, which is supported by prior studies [3,10].

Strengths of the study include incorporating a statewide, population-level surveillance system over a single institutional study, increasing study generalizability. The study had several limitations. Small sample sizes precluded our ability to explore variables in a multivariate analysis, possibly limiting statistical power to detect differences. Although the case report forms are standardized across institutions, variability in documentation practices and interpretation of clinical data across institutions and providers may have influenced data quality on case report forms. The dataset did not include a timeline of symptom onset, which may have impacted the correct classification of EOGBS versus LOGBS. Variables such as maternal allergies to penicillin and the timing of maternal antibiotic administration were not included in the dataset. Maternal history of penicillin allergy may affect prophylactic antibiotic choice. Specific data regarding dates of antibiotic administration and time was not available, so the impact of precipitous delivery on the number of maternal antibiotic doses is unknown. We relied on clinical and microbiology data to establish a diagnosis of meningitis. However, antibiotic initiation prior to CSF culture collection affects the ability to identify GBS on culture, which may account for the false sterilization of CSF cultures [15]. Future research into factors associated with LOGBS and EOGBS should utilize larger sample sizes and time-specific data to adjust for confounding variables.

## Conclusions

Our study demonstrated differences in factors associated with EOGBS and LOGBS. Strategies for EOGBS prevention include maternal antibiotic prophylaxis and screening. The transmission and pathogenesis of LOGBS are likely different than EOGBS, creating a need for different preventative strategies. Potential mechanisms to prevent LOGBS include maternal vaccination due to maternal antibodies that provide long-term protection from a diverse set of GBS infection sources.
